# BioDB extractor: customized data extraction system for commonly used bioinformatics databases

**DOI:** 10.1186/s13040-015-0067-z

**Published:** 2015-10-28

**Authors:** Rajiv Karbhal, Sangeeta Sawant, Urmila Kulkarni-Kale

**Affiliations:** Bioinformatics Centre, Savitribai Phule Pune University, Ganeshkhind, Pune, 411007 Maharashtra India

**Keywords:** Bioinformatics, Biological databases, Customized data retrieval, Data integration, Database cross-linking

## Abstract

**Background:**

Diverse types of biological data, primary as well as derived, are available in various formats and are stored in heterogeneous resources. Database-specific as well as integrated search engines are available for carrying out efficient searches of databases. These search engines however, do not support extraction of subsets of data with the same level of granularity that exists in typical database entries. In order to extract fine grained subsets of data, users are required to download complete or partial database entries and write scripts for parsing and extraction.

**Results:**

BioDBExtractor (BDE) has been developed to provide 26 customized data extraction utilities for some of the commonly used databases such as ENA (EMBL-Bank), UniprotKB, PDB, and KEGG. BDE eliminates the need for downloading entries and writing scripts. BDE has a simple web interface that enables input of query in the form of accession numbers/ID codes, choice of utilities and selection of fields/subfields of data by the users.

**Conclusions:**

BDE thus provides a common data extraction platform for multiple databases and is useful to both, novice and expert users. BDE, however, is not a substitute to basic keyword-based database searches. Desired subsets of data, compiled using BDE can be subsequently used for downstream processing, analyses and knowledge discovery.

**Availability:**

BDE can be accessed from http://bioinfo.net.in/BioDB/Home.html.

## Background

The data available for biological systems are diverse in nature and include various types such as sequences, structures, expression data, interactions, pathways, systems data, phenotype(s) and population data [1]. The rate at which the data are generated has increased exponentially due to technological advances in the fields of genomics, transcriptomics, proteomics, structural genomics, metabolomics, systems biology etc. [2]. The changing nature of studies in areas such as personalized genomics and translational medicine has further accelerated the pace of data generation resulting in greater volumes of data [3, 4]. As the biological data constitutes an important component of ‘Big Data’, it demands novel solutions to curate, compile, organize, archive, query and analyse in order to deal with three Vs, namely, volume, velocity and variety [2, 5–7]. Various types of primary data along with annotations continue to be useful for processing, analysis and interpretation of inherently complex high throughput data so as to generate higher order information and knowledge. The existing databases archiving molecular data are built around focused themes and offer relevant annotations. Variety of derived data are built around each type of primary data and cross-linking within as well as across primary and derived databases exist to a large extent [8, 9].

The tools that facilitate extraction of the data and integration of diverse data types are therefore the need of the hour. A number of online as well as offline tools and servers are available for accessing and retrieving large amounts of data from public domain resources. Some of the tools are highly resource-specific or cater to specific data types while a few tools facilitate access to data from different resources/databases through a single interface. NCBI E-utilities, for an instance, provide customized data extraction utilities for various databases available at NCBI [10]. These utilities require generation of URL by the user in a format specific for respective databases either manually or by writing scripts. Although, multiple URLs can be generated, there are limits on the number of URL requests per second (three per second) as well as per day (100 requests per day), which can be submitted through an IP address. More over the users are required to limit large jobs on weekends or between 9:00 PM and 5:00 AM Eastern time during weekdays. PDB Goodies is another example of resource-specific server that offers various tools for retrieving and mining the data in Protein Data Bank ([11], http://dicsoft2.physics.iisc.ernet.in/pdbgoodies/inputpage.html. On the other hand, BioDownloader [12], PBIL [13], SIR [14] BioMart [15] etc. are some of the examples of tools catering to multiple resources.

BioDownloader is a program to download a data set from FTP and HTTP servers of different resources such as NCBI FTP server, PDB FTP server etc. [12]. BioDownloader is very useful for downloading large datasets from remote servers. Selective extraction of desired data files / entries from specified resources, however, is not supported by this program. PBIL provides integrated access to nucleotide and protein sequences from ENA (EMBL-Bank), GenBank and SWISS-PROT databanks and supports sequence/structure analysis services e.g. BLAST, GOR for downstream processing [13]. It may be noted that PBIL does not permit extraction of other subsets of data from entries of respective databases. SIR is a tool written in python for indexing and searching flat files from different databases [14].

BioMart is a generic scalable system which provides unified access to biological data archived in the diverse and geographically disparate biological databases through a central portal [15, 16]. It supports an open ended architecture that facilitates addition of new databases. The system is designed and widely used with genomic and data generated using high throughput technologies.

It may be summarized that available resource-specific tools as well as the tools that integrate multiple resources cater to various needs of data downloads and extraction. These tools are comprehensive and facilitate several types of queries. Some of the servers, however, have specific limitations. They facilitate either (a) downloading of limited number of entries from a single source in a session or in a day [for example, NCBI E-utilities]; or (b) downloading of complete entries only [for example, Biodownloader]; or (c) searching and retrieval of only limited sub sets of data. Thus, utilities for customizable and in-depth retrieval of subsets of data from entry/ies of databases, may complement and enhance the existing tools.

As an initial step to reach this goal, a portal named BioDB Extractor (BDE) has been developed that makes the existing search utilities more effective and productive towards customized data extraction. BDE does not duplicate keyword based searches which are extensively supported by the search engines made available for the respective databases and focuses on facilitating need-based data extraction in interactive and batch modes. It is intended to assist the user to extract subsets of data on the fly directly from the respective resources, avoiding duplication of data on local machines.

## Methods

The current version of BDE includes data extraction utilities designed for five widely used primary databases such as European Nucleotide Archive (ENA: EMBL-Bank) [17, 18], UniProtKB/Swiss-Prot [19], Protein Data Bank (PDB) [20], Kyoto Encyclopedia of Genes and Genomes [KEGG] [21] and DrugBank [22]. Dictionary of Protein Secondary Structure (DSSP), a widely used method for assignment of secondary structures has also been integrated in BDE [23].

BioDB Extractor is a web-based system developed on the MS Windows operating system. Apache HTTP Server (Version-2.2.21) is used as a web server. The scripts are written in Perl (v5.14.2). Modules of Perl viz., ‘Library for WWW in Perl (LWP)’ and ‘WWW-Mechanize’ are used to connect to respective databases for the purpose of data extraction and parsing. HTML, JavaScript, PHP are used for development of web interfaces. The server can be accessed using the URL http://bioinfo.net.in/BioDB/Home.html.

The customized data extraction utilities of BDE make use of web services based on Representational State Transfer (REST), Web Services Description Language (WSDL) and Simple Object Access Protocol (SOAP) provided by respective databases [24, 25].

These are listed in Table 1.

**Table 1:** Databases and corresponding web services

Database Name	Web service type: URL
NCBI	E-utility Web Service (SOAP & WSDL): http://www.ncbi.nlm.nih.gov/books/NBK43082/
EMBL/EBI	EMBL-EBI Web Services-REST: http://www.ebi.ac.uk/Tools/webservices/tutorials/02_rest
UniprotKB	Programmatic access services-REST: http://www.uniprot.org/faq/28
PDB	RCSB PDB RESTful Web Services- REST: http://www.rcsb.org/pdb/software/rest.do
KEGG	REST-style KEGG API: http://www.kegg.jp/kegg/rest/keggapi.html

The typical process flow of utilities in BDE is given below. A script in BDE:

- parses the input provided by the user
- connects to the relevant entry/ies in the respective database
- parses the entry/ies to extract user-specified record(s)
- generates and returns the output through user interface.

## Results and discussion

### Results

Along with the growth in volumes and varieties of molecular data, numbers and sizes of molecular databases are ever increasing. This is evident from the fact that the first database issue of NAR published in the month of January 1999 reported 202 databases [26] whereas the most recent issue (January 2014) catalogues 1594 databases sorted into 15 categories and 41 subcategories [27]. In this issue there are reports of 58 newly developed databases & 123 reports of previously developed databases with significant updates. These curated public domain databases cater to the needs of users in the area of Life Sciences as well as other specialized areas.

Searching/browsing these databases and generating curated datasets is an essential prerequisite for processing, analysis and interpretation. Text-based search engines such as SRS [28] and Entrez [29] are extremely useful and permit use of simple and comprehensive search strategies to generate desired datasets comprising of relevant complete entries/sequences from the database. Extraction of subsets of data, where only a part of the data/sequence are to be retrieved from complete entries of interest, however, requires an additional level of processing, which is not necessarily available directly from the above-mentioned search engines. For example, it is possible to search the nucleotide sequence entries based on the 63 different feature keys of the feature table; however, the search engines do not support extraction of data from individual features. This makes it necessary for the user to download complete entry/feature table, write scripts to parse these and extract the desired feature/s data. It becomes all the more laborious when users need to search and extract subsets of data from a large number of entries, requiring batch processing.

**Fig. 1:**
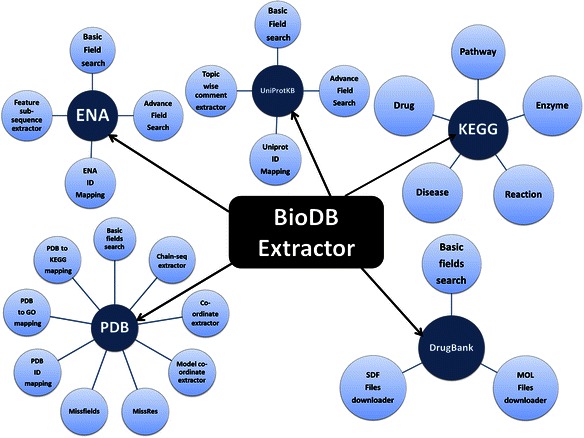
A schematic summary of BioDB Extractor (BDE) showing the available database resources (Dark blue circles) and related utilities (light blue circles) for each resource

**Fig. 2:**
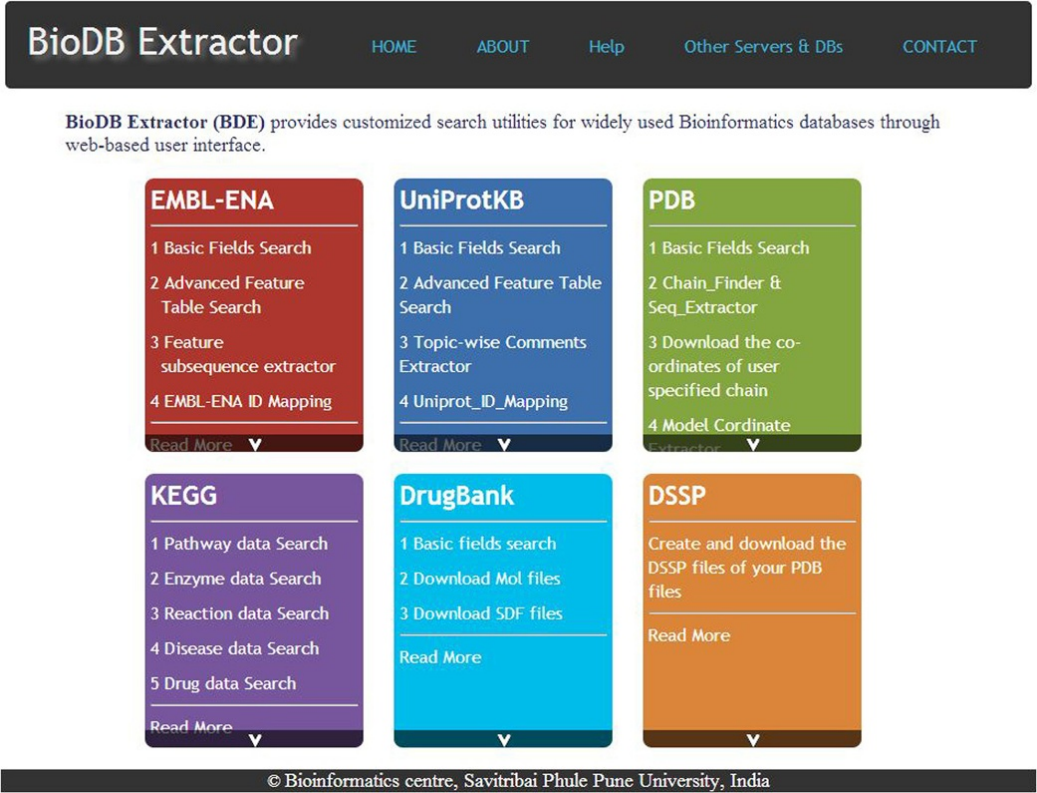
A snapshot of the Home Page of BioDB Extractor (BDE)

BDE attempts to address this need for searching and extraction of subsets of data from few widely used databases by providing highly customizable data extraction from various fields of a single entry as well as multiple entries in a batch processing mode. Schematic summary of BDE utilities is shown in Fig. 1. BDE provides a single platform with a user-friendly interface and eliminates the need for downloading complete entries and writing scripts to parse them for data extraction; thus facilitating both, expert as well as novice users (Fig. 2).

Some of the common features of all utilities in BDE are:

1. All utilities accept input in the form of ID codes/accession numbers of entries of respective databases. If an erroneous/invalid accession number is provided to BDE, it returns the message “Invalid database ID”.
2. All utilities support data extraction from single entry as well as multiple entries (batch processing).
3. All utilities involving selection of fields/features by the user display complete lists of available fields/features/qualifiers on the screen.
4. All utilities generate customized data sets for user-specified database entries that can be directly used for various types of analyses.

In addition to these features, distinction of BDE lies in meta-customization specific to individual databases.

The database specific utilities in BDE are described below.

### ENA (EMBL-Bank) Nucleotide sequence database

ENA (http://www.ebi.ac.uk/ena/), GenBank (http://www.ncbi.nlm.nih.gov/genbank/) and DDBJ (http://www.ddbj.nig.ac.jp/) are the nucleotide sequence databases that are members of the International Nucleotide Sequence Database Collaboration (INSDC) [30]. The data is exchanged among these databases on a daily basis. BDE provides two customizable data extraction utilities for the ENA (EMBL-Bank) database viz. Basic Fields Search and Advanced Feature Table Search.

### Basic fields search

The Basic Field Search utility accepts user input in the form of list of ENA (EMBL-Bank) accession numbers followed by selection of data fields by the user to search the database and display the subsets of extracted data from the user specified entries. The list of accession numbers may be provided in an interactive mode or by uploading a text file.

### Advanced feature table search

The feature table (FT) block is an important section in a nucleotide sequence entry. Currently 63 standard feature keys have been listed by INSDC (30) such as CDS, Exon, GC_signal, Intron, Mobile_element, mRNA, Operon, TATA_signal, 5′UTR, −35_signal etc. Advanced Feature Table Search utility is a unique utility of BDE which allows the users to selectively extract one or more features from the feature table block from an entry / a set of entries. Typical format of feature table consists of a feature key (a single word or abbreviation indicating the functional group/feature), location (position/s or boundaries of the feature in the sequence) and qualifiers (auxiliary information about a feature). The format and contents of feature tables are identical for equivalent entries in all the member databases of INSDC (The DDBJ/ENA/GenBank Feature Table Definition, Version 10.2 November 2012). The Advanced Feature Table Search utility also accepts list of accession numbers as input from the user and allows selection of desired feature/s for data extraction. It produces an output containing the annotations available in the qualifiers of selected feature key/s in the user-specified entry/ies. Any cross-references (db_xref) to other databases, if available for the selected feature, are also displayed in the output in the form of ID Code/s or accession number/s, with links to the respective records in reference databases. Additionally, BDE also provides cross-links to other relevant databases for some of the qualifiers which are not explicitly available in the original ENA (EMBL-Bank) database entries. For example, the qualifiers such as ‘/EC_number’, ‘/gene’, ‘/protein_id’, ‘/locus_tag’ etc. provided under the features such as CDS and gene, if available, are cross-linked to KEGG, Brenda, enzyme@ExPASy and other relevant databases. The mapping of database accession numbers and cross linking is carried out by using the base URL of the database and appending the appropriate tags (referring to the entry in the database) to generate the URLs to relevant entries. During the extraction of qualifiers of user-specified features from an entry, a feature may appear more than once in the entry. In these cases, the queried qualifiers of each feature are grouped and displayed in a feature-wise manner. For example, several entries contain multiple coding sequences (CDS feature) and each CDS has multiple qualifiers such as /gene, /EC_number, /product, /protein_id etc. BDE displays the data in qualifiers of each CDS in a segregated manner. This enables the user to view and save the data of each feature in a logically organized and user-friendly format.

### Feature subsequence extractor

For most of the features in a typical nucleotide sequence entry, the range of the subsequence which represents the feature is documented. This applies to features such as CDS, exons, introns, mRNA etc. as can be seen in the examples below:

**Table:** 

FT CDS	complement(join(1855..2361,2403..2426))
FT exon	complement(1855..2361)
FT mat_peptide	complement(join(1858..2361,2403..2426,2459..2965))
FT mRNA	join(<3437..4757,4915.. > 5057)

It is often useful to extract feature-specific fragment/subsequence for further analysis. The ‘Feature Subsequence Extractor’ utility is available in BDE for this purpose. Users can select the feature/s for subsequence extraction. The subsequence/s of specified range corresponding to the selected feature/s is generated and displayed on the screen. Unlike the region/range selection utility in Entrez at NCBI, BDE facilitates the subsequence extraction from multiple features at a time.

The customized data extraction utilities for ENA (EMBL-Bank) database in BDE, thus, are a step forward towards more effective and improved extraction of data available in the databank and help in compilation of desired datasets for downstream processing. To the best of our knowledge, existing search and retrieval utilities of the respective INSDC databases do not facilitate customization of this level unless complete entries are downloaded and scripts are written to parse and extract the desired data set/s.

### Protein sequence database (UniprotKB/Swiss-Prot)

UniprotKB/Swiss-Prot ([19], http://www.uniprot.org/) is a highly reliable and comprehensive resource for protein sequences due to its features such as rich annotations, manual curation, minimal redundancy and extensive integration with other databases. BDE provides ‘Basic Search Utility’ for customizable search/extraction of subsets of data from all the 19 fields that are available in a typical entry of UniprotKB/Swiss-Prot database (UniprotKB User Manual Release 2013_08 of 24-Jul-2013). This utility is similar to the Basic Field Search utility for ENA (EMBL-Bank) database described above.

The ‘Advanced Annotations Extractor’ in BDE allows extraction of single or multiple features from the Feature Table (FT) block and Comments section in the entries of UniprotKB/Swiss-Prot database. This section of a typical SwissProt entry contains rich annotations for proteins. FT block has 40 different types of feature keys while the records in the Comments section, designated with line id ‘CC’, contains 29 classes of topic-wise annotations.

### ID mapping utility

Searching and extracting relevant data for desired biological object/system from different databases is often a necessity for data analysis, interpretation and knowledge generation. It demands accessing the appropriate entries for the same object/system in different databases. This facility provided by the ‘ID mapping’ service available for UniProtKB, is an important and highly useful service. It finds equivalent data entries/sets from 96 databases. Using this service, user-specified ID code/s from reference database can be mapped on to equivalent ID code/s from only one target database at a time. BDE offers a value added and extended ID mapping utility, which (a) facilitates simultaneous mapping of UniProtKB/SwissProt ID codes to multiple target databases in one step and (b) provides a total of 109 most widely used target databases to which ID mapping can be done. It is planned to extend this utility to map ID codes of any database to all other databases considered here.

### Protein structure database (PDB)

PDB is a repository of 3D structural data of biomolecules (http://www.rcsb.org/). BDE provides several utilities for (a) extracting and downloading subsets of data, (b) identifying missing fields / amino acid residues of protein chains in PDB files and (c) crosslinking PDB entries to other databases.

The Basic Fields Search utility for PDB facilitates extraction and downloading of data from desired fields from a single or multiple PDB entries specified by user. A typical entry in PDB may contain a total of up to 48 different data fields (Protein Data Bank Contents Guide: Atomic Coordinate Entry Format Description Version 3.30; Nov. 21, 2012). Out of 48 defined fields, 16 fields are mandatory for each PDB entry and 34 are optional (http://www.wwpdb.org/documentation/format33/sect1.html). In view of variations in the contents found in PDB entry/ies due to optional fields, BDE provides ‘MissFields’, a utility that identifies and lists the missing fields in PDB entries, i.e. fields for which data is/are not available.

### Chain_Finder & Seq_Extractor

Given the PDB ID code/s as input, the Chain_Finder utility finds and lists the number of chains in specified entry/ies and lists the number and chain indicators/Ids of polypeptide chain/s in the output. The Seq_Extractor utility of BDE allows users to extract and download FASTA formatted sequence/s of one or more desired chain/s by providing PDB IDs as input. A similar utility is available at RCSB PDB server (http://www.rcsb.org/) but it extracts the sequences of all the chains in the entry/ies and does not provide an option to select one/more chains of interest.

### Coord_Extractor

This utility of BDE facilitates extraction of atomic coordinates of desired chain/s from a set of PDB entries. An option is also provided to save/download the PDB-formatted coordinates of all/selected chain/s from each PDB entry either as a single file or in separate files for each chain. The multiple coordinate files thus saved are made available for downloading in zipped format.

For the protein structures solved by NMR spectroscopy, multiple models representing various conformers of the proteins are available in PDB entry/ies. The Model_coordinate_Extractor utility of BDE caters to such entries by facilitating users to selectively download the co-ordinates of one or more models from the available set. It also extracts and displays the information for the best available model (specified in corresponding PDB entry) so that the user may selectively extract the coordinates of the same.

### MissRes

Very often the coordinates of some of the amino acid residues of protein chain/s are not reported in the PDB entries (missing residues) either due to highly flexible nature of corresponding segments of the polypeptide and/or low resolution of the electron density maps. Such incomplete co-ordinates data may be problematic in certain applications such as homology modeling (when being used as a template). In a typical PDB file, these missing residues are listed in REMARK 465 (wwPDB Processing Procedures and Policies Document, December 2012 Version 2.6). In mmCIF format _pdbx_poly_seq_scheme contains the data of missing residues denoted as ‘?’. The ‘MissRes’ utility of BDE can be used for finding and extracting a list of missing residues in user-specified chain/s of PDB entry/ies. This utility employs two approaches to identify the missing residues and both are made available to the user simultaneously. In the first approach, the information in REMARK 465 records from PDB files and/or _pdbx_poly_seq_scheme from mmCIF files is used to generate the list of missing residues. In addition to amino acid residues, the reported missing nucleic acids in the DNA/RNA chains are also listed in the output. In the second approach, the amino acid sequence of a polypeptide chain is extracted from the coordinate section of PDB file and aligned with the sequence available in the SWISSPROT entry of the same protein (considered as reference sequence) so as to identify the missing amino acid residues for which no co-ordinates are available. The alignment as well as list of missing residues are displayed in the browser window and are also made available for downloading. The pairwise alignment is carried out at the backend, using the EMBOSS Needle program available at the EMBL-EBI (http://www.ebi.ac.uk/Tools/psa/emboss_needle/) [31, 32].

### ID mapping

The purpose of this utility is to map the PDB ID codes to 109 public domain primary and derived databases belonging to various categories such as Sequence databases (GenBank, EMBL-Bank, DDBJ, UniProtKB, The International Protein Index (IPI)); Protein-protein interaction databases (DIP, IntAct, STRING, MINT), protein family and domain databases (Pfam, PRINTS), etc. [19, 33–42]. To the best of our knowledge, such a utility to map the PDB IDs directly is not available at the RCSB PDB server. Mapping of PDB IDs enables users to access relevant data across several databases in simplified and efficient manner.

### Sec_Struct_Extractor

BDE provides this tool to assign secondary structures to amino acid residues in a protein on the basis of the available 3D structure. The stand-alone executable of the DSSP program is used at the back-end for this purpose (http://swift.cmbi.ru.nl/gv/dssp/) [23, 43]. Sec_Struct_Extractor accepts standard PDB ID code/s as input as well as allows users to upload locally saved PDB files (e.g. predicted/ modeled structures) for the purpose of computation of secondary structures. This tool avoids the absolute necessity to download the PDB entry files by the users.

### PDB to GO mapping

Gene ontology (GO) terms provide structured controlled vocabularies (ontologies) that describe gene products in terms of their associated biological processes, cellular components and molecular functions in a species-independent manner. To link the structural data with the function, it is often necessary to map the PDB IDs to GO terms, which are widely used for annotation and classification purpose. BDE provides the utility to map PDB IDs and corresponding chain IDs to GO terms. The summary page of each PDB entry contains a hyperlink to the GO terms (in the section: External Domain Annotations). This hyperlink is used to access the relevant GO terms which are then displayed in the results. Mapping of PDB IDs to GO terms enables users to extract relevant annotations for each chain in simplified and efficient manner which can be used subsequently for analysis and interpretation.

### PDB to KEGG mapping

This utility in BDE can be used to map the PDB IDs directly on the KEGG metabolic/signaling pathway/s in which the queried enzyme/s is/are involved. Such a mapping helps scientists in establishing correlations between the biochemical and cellular level functions of enzymes. The mapping to KEGG pathway is done on the basis of EC number of the enzyme. The output lists the names of mapped pathway/s and also displays the pathway diagrams in which EC number of queried enzyme is highlighted.

### KEGG resource

**Fig. 3:**
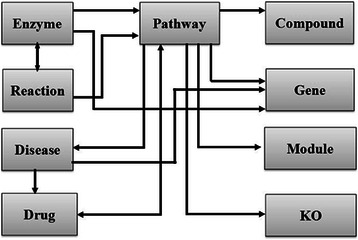
Schematic representation of the utilities available for databases available at the KEGG resource. Arrows connecting the databases indicate directionality of searching the databases using BDE utilities. Unidirectional arrows indicate search of target database (to which arrow-head is pointed) using keywords/data types from the resource database. Bidirectional arrows indicate utilities that facilitate two-way searches of databases

KEGG is a major resource for metabolic and signaling pathways and related data as well as analysis tools [21]. BDE provides 27 utilities that facilitate customized search of various types of data across the constituent datasets available in KEGG. These utilities also support batch retrieval and enable an easy access to data types at various levels ranging from genes to pathways. To name a few, utilities are available for searching and retrieval of list/s of enzymes involved in different pathways, chemical reactions catalyzed by the specified enzyme/s, environmental factors responsible for diseases etc. Such data sets are a prerequisite for comparative studies, systems biology and pathway engineering. Schematic summary of KEGG utilities is shown in Fig. 3.

As indicated in the schematic diagram the KEGG constituent databases can be searched across the resource in several ways. Some of the search utilities can be used in bidirectional manner while others are unidirectional. For instance, the user can search the drug database using pathway data as a query and vice a versa, i.e. pathways can be searched using drug data as a query. On the other hand, gene names can be used to search pathways database but gene search using pathway names as a query is not supported. A summary of the utilities is provided in Table 2.

**Table 2:** KEGG Utilities

Data type	Utilities available for data search and extraction
Pathway	Get Genes by Pathway
	Get Diseases by Pathway
	Get Drugs by Pathway
	Get Compounds by Pathway
	Get KO pathway
	Get Module by pathway
Enzyme	Get systematic name of the enzyme
	Get class of enzyme
	Get reaction/s by enzyme
	Get pathway by enzyme
	Get genes by enzyme
Reaction	Get the definition of Reaction/s
	Get the Equation of Reaction/s
	Get the Enzyme/s by Reaction
	Get pathway by Reaction
Disease	Get the category of disease
	Get pathways by disease
	Get genes by disease
	Get Environmental factors responsible for Disease
	Get markers of Disease
	Get list of drug used for treatment of disease
Drug	Get Formula of drug
	Get Exact mass of drug
	Get Molecular weight of drug
	Get principle activity of drugs
	Get Source of drug
	Get target of drug

The unique feature of BDE is that it provides customized data retrieval utilities that are not available explicitly at the KEGG resource. For example, using BDE, it is possible to extract different data types (pathway specific genes, diseases, drugs, compounds, module and KO) for multiple organisms and multiple pathways simultaneously. In contrast, KEGG utilities in the present form facilitate search of single pathway of a single organism at a time.

### DrugBank

DrugBank is the database of small molecules, drugs and drug targets. The utilities provided by BDE for this database are:

### Basic fields extractor

BDE provides Basic Fields Extractor, which is a customized data retrieval utility for fields in a DrugBank data entry such as Name, Accession Number, CAS number, Synonyms, IUPAC Name, SMILES strings, Chemical Formula, InChI (International Chemical Identifier) etc.

Scientists are often required to download structures of several small molecules/drugs in a single step, for applications involving high throughput screening or QSAR. The search engine of DrugBank does not support such a batch processing utility. In view of this, BDE provides two batch retrieval and downloading utilities, through which users can download structures of drugs/small molecules in MOL or SDF file formats.

### BDE: usability in workflow mode

**Fig. 4:**
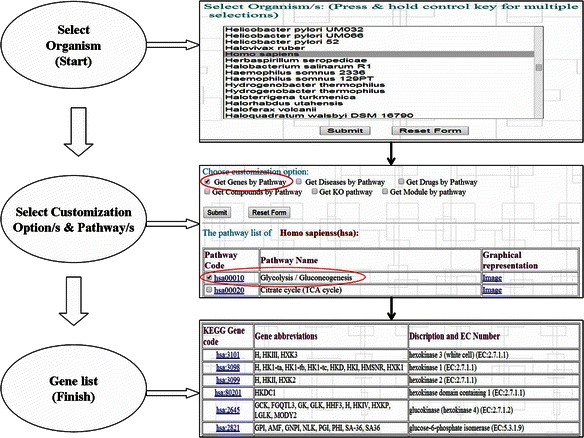
A simple query and the corresponding processes in BDE are shown. This example illustrates how to extract all the genes of a specific pathway/s for a given organism/s

**Fig. 5:**
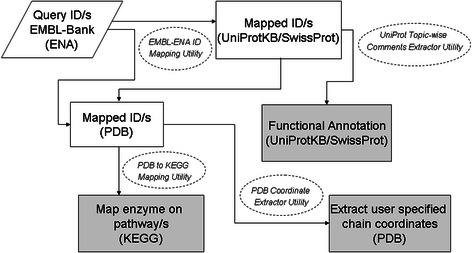
A diagram depicting usage of various utilities and databases in BDE. With ENA (EMBL-Bank) as a starting point, four utilities in BDE (shown as ovals with dashed border) leads to extraction of three different data sets (shown as gray boxes) from three independent databases. The arrows indicate how BDE utilities could be combined to generate implicit and explicit workflows

**Fig. 6:**
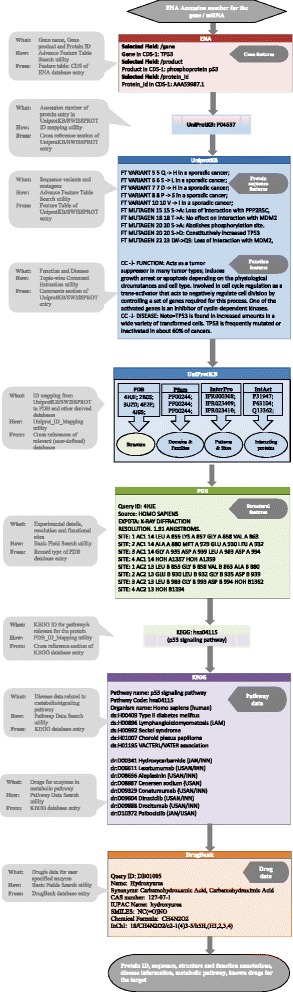
An illustration of how BDE can be used to extract the various types of data for a drug target starting with ENA accession number of the gene. As shown in the figure, users can navigate through and extract the gene features (CDS), Protein ID (from UNIPROTKB/SWISSPROT), protein sequence, structural and functional features, pathway data and drug molecules available for the desired protein. The gray callouts in left half of the figure document the search/query details for each query of BDE and the boxes in the right half show the data obtained at various levels

The BDE homepage (Fig. 2) provides extensive help and documentation on how to use this resource. An example of how a simple query can be performed to extract desired data is provided in Fig. 4. The utilities designed for various databases can be combined to generate customized workflows. An illustration of how implicit and explicit workflows can be built is shown in Fig. 5. For example, with ENA (EMBL-Bank) as a starting point, the user could choose to employ any of the four implicit workflows or their combinations to extract data of one or all the four levels viz sequence, structure, function and pathways. Figure 6 illustrates how BDE can be used to extract the different levels of data from the relevant databases in a workflow approach using an example of P53 gene. As can be seen in this figure, the user needs to provide only the accession number of the ENA entry of the gene (P53) and navigate through the various utilities to extract protein product ID, protein sequence, structure and functional features along with the disease data. Further, the metabolic pathway data and the available drug data can also be extracted. This workflow thus, facilitates the researcher to access multi-layered data for the gene/protein of interest. The results may be further utilized for validating the drug target, development of new drugs, exploring alternative strategies for drug development with other potential targets in the same metabolic pathway, etc.

### Efficiency and benchmarking of BDE

The benchmarking statistics of the some of the utilities in BDE is given in Table 3. However, the time required for processing of data using BDE would depend on various factors such as number of entries of a database to be parsed, size of data, number of fields and features selected for extraction and the internet connectivity etc. The way BDE is designed, maintaining local versions (full or partial), of the included databases is not necessary at both, the server and client sides. The queries are processed on-the-fly and results are made available to the user. User gets the benefit of accessing the data from the latest available version of the respective database directly.

**Table 3:** Processing time statistics of some utilities in BDE

Database	Type of input data: (Number of IDs)	Size of data being parsed (Mb)	Utility	Time (seconds)^a^
EMBL-ENA	Genes: 250	12.4	Basic Fields Search (All Fields are selected)	80
			Advanced Feature Table Search	95
	Genomes: 100 (Viral & bacterial)	137	Basic Fields Search (All Fields are selected)	120
UniprotKB	Proteins: 250	2	Advanced Feature Table Search	30
	Proteins: 250	2	Topic-wise Comments Extractor	30
PDB	Structures: 250	200	Chain_Finder & Seq_Extractor	120
	Structures: 250	200	PDB to GO Mapping	100
KEGG	Organisms: 10	-	Customized searches for Pathway data (GENE)	45
DrugBank	Drugs: 250	14	Basic Fields Search (All Fields are selected)	70

^a^:This column lists the durations required for the processing of each query, beginning with input of ID list to the server, up to the display of results on the user interface

### Discussion

BDE provides a simple and easy to use interface with several utilities for customized data search and extraction. It is efficient and eliminates the need for downloading complete entries and script-writing for parsing and data extraction. It supports batch processing for multiple entries specified by users. The target user group of BDE may include life science researchers/domain experts as well as trained computational biologists & bioinformaticians. With its simple and informative user interface, BDE also has the potential to be a useful tool for beginners and novice users interested in generating curated data sets comprising of subsets of data. Future versions of BDE would cover wider range of primary and derived databases along with suitable data extraction utilities.

In the era of Big Data in biology, which is full of diversity, threading various layers of primary and derived data using hard as well as soft links/connections is the need of the hour so as to facilitate knowledge discovery. In this context, it is imperative to harness and strengthen the existing resources/database through development of a network that traverses within and across the individual layers of molecular data. For example, a gene could be used as a basic unit to connect genomic data to systems data via protein sequence/s, three-dimensional (3D) structure/s, functions, interactions, pathways etc. These types of connections, to a large extent, exist in the currently available primary databases and are referred to as ‘hard links’. However, the process of knowledge discovery demands that beyond hard links, implicit logical connections across datasets from diverse databases need to be drawn. These may be referred to as ‘soft links’ and technology/ies to capture soft links need/s to be in place. A schema for manifestation of soft links in the existing and forthcoming databases would provide new insights and perspectives for life science research. It will truly harness the power of Bioinformatics in data driven discovery. These soft links could be dynamically established using property/ies such as homology, structural, functional similarities, membership to a certain biological process etc. To achieve this gigantic task, a multi thronged approach needs to be adopted. BDE may be viewed as a first step towards the foundation of a large effort for creating soft links.

## Conclusions

BDE has been developed with the objective of providing a single platform for customizable data extraction from some of the major biological databases. The on-the-fly processing of user queries eliminates duplication of data on local machines and facilitates access to the most up-to-date version of the parent database. It adds to the capabilities of the existing tools and servers for data extraction and analysis.

## Abbreviations

BDE: BioDB Extractor
ENA: European Nucleotide Archive
PDB: Protein Data Bank
KEGG: Kyoto Encyclopedia of Genes and Genomes
DSSP: Dictionary of Protein Secondary Structure
INSDC: International Nucleotide Sequence Database Collaboration
