# Erratum to: BioDB extractor: customized data extraction system for commonly used bioinformatics databases

**DOI:** 10.1186/s13040-016-0081-9

**Published:** 2016-02-02

**Authors:** Rajiv Karbhal, Sangeeta Sawant, Urmila Kulkarni-Kale

**Affiliations:** Bioinformatics Centre, Savitribai Phule Pune University, Ganeshkhind, Pune, 411007 Maharashtra India

After publication of this article [1], the authors noticed that Fig. 6 (Fig. 1 here) had been published incorrectly. The following sections were omitted from the Figure: ‘UniProtKB’, ‘PDB’, ‘KEGG’, ‘DrugBank’. The revised version of Fig. 6 (Fig. 1 here) has been updated in the original article [1] and is also located below:

**Fig. 6:**
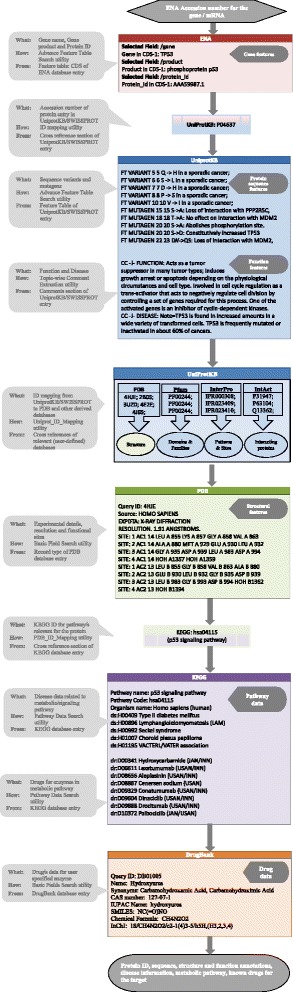
ᅟ
